# Task-dependent fMRI decoder with the power to extend Gabor patch results to Natural images

**DOI:** 10.1038/s41598-020-58241-x

**Published:** 2020-01-28

**Authors:** Yoshiaki Tsushima, Yasuhito Sawahata, Kazuteru Komine

**Affiliations:** 1 Center for Information and Neural Networks, National Institute of Information and Communication Technology, 3-5, Hikaridai, Soraku-gun, Seika-cho, 619-0289 Kyoto Japan; 2 Science & Technology Research Laboratories, Japan Broadcasting Corporation (NHK), 1-10-11 Kinuta, Setagaya-ku, 157-8510 Tokyo Japan

**Keywords:** Human behaviour, Visual system

## Abstract

Scientists are often asked to what extent a simple finding in a laboratory can be generalized to complicated phenomena in our daily lives. The same is equally true of vision science; numerous critical discoveries about our visual system have been made using very simple visual images, such as Gabor patches, but to what extent can these findings be applied to more natural images? Here, we used the fMRI decoding technique and directly tested whether the findings obtained with primitive visual stimuli (Gabor patches) were applicable to natural images. In the fMRI experiments, participants performed depth and resolution tasks with both Gabor patches and natural images. We created a fMRI decoder made from the results of the Gabor patch experiments that classified a brain activity pattern into the depth or resolution task, and then examined how successful the task-dependent decoder could sort a brain activity pattern in the natural image experiment into the depth or resolution task. As a result, we found that the task-dependent decoder constructed from Gabor patch experiments could predict which task (depth or resolution task) a participant was engaged in the natural image experiments, especially in the V3 and middle temporal (MT+) areas of the brain. This is consistent with previous researches on the cortical activation relating to depth perception rather than perceptual processing of display resolution. These results provide firm evidence that fMRI decoding technique possesses the power to evaluate the application of Gabor patch results (laboratory findings) to the natural images (everyday affairs), representing a new approach for studying the mechanism of visual perception.

## Introduction

**Figure 1:**
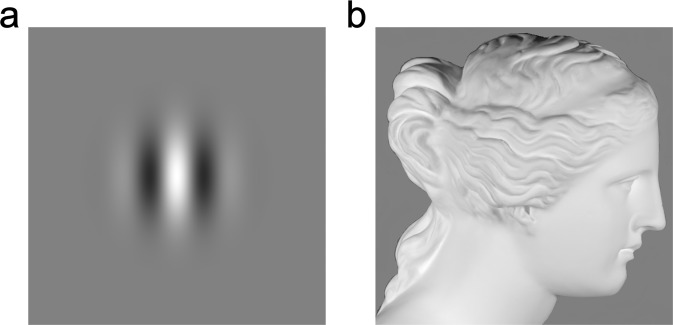
(**a**) Gabor patch. (**b**) Example of a natural image (sculpture) (drawings were created by Y.S. and Y.T., and modified using Adobe Illustrator CC.).

To examine puzzling phenomena in our daily lives, scientists generally conduct simple and disciplined experiments in a laboratory. For example, Pavlov used a dog and a bell in his laboratory to determine why his dog salivated when he arrived home^1^. However, researchers are often asked how relevant the results found under such controlled conditions are for explaining complicated matters in the real world. In vision science, a great number of researchers have tried to explore humans’ visual experiences. To investigate the visual mechanism, most vision scientists have conducted psychophysical experiments using very primitive images such as Gabor patches (Fig. 1a), which accurately reflect the receptive field of neurons in the primary visual cortex^2^.

Although numerous important discoveries about our visual system have been made using Gabor patches, it is natural to ask whether these findings can be applied to more natural images (Fig. 1b)? There has been some difficulty in proving this, because a natural image is not usually quite as simple as a Gabor patch. A natural image generally includes more complicated visual features, such as shape or texture, which are not easily expressible with one Gabor patch. In addition, our perception of natural images might be more strongly influenced by higher cognitive functions such as memory^3^. For instance, victims of a tsunami might look at sea waves in a different way to others. Taken together, we have a serious problem whereby we cannot use a natural image itself to rigorously investigate the visual mechanism of processing such an image. How can we overcome this long-standing dilemma?

Recently, several functional magnetic resonance imaging (fMRI) techniques have provided us with unique research methods for investigating the mechanism of processing a natural image^4–10^. For example, Miyawaki *et al*. demonstrated the possibility of reconstructing complex visual images from the voxel patterns of fMRI activities for a simple visual image such as a black-and-white checkerboard^8^. The basic idea of such a visual decoding is that the neural processing of a complex image (e.g., a natural image) is equivalent to the assembled mass of the neural processing of a simple image. Furthermore, from research on primate inferotemporal cortex researches, it is known that images of complex objects can be reduced to critical features that generally consist of less complex parts of the object images^11–15^. That is to say, we might be able to uncover common ground between neural information processing with primitive visual stimuli (Gabor patches) and one with complex images (natural images) by such fMRI methods. Could this kind of fMRI decoding technique help us to solve the long-standing problem described above?

**Figure 2:**
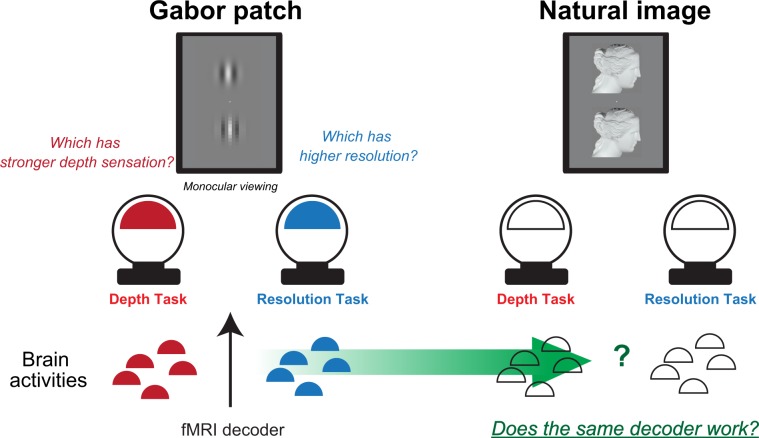
Research paradigm: firstly, participants performed both Depth and Resolution tasks with a Gabor patch (left)^19^ under fMRI observation. We created a task-dependent decoder from the Gabor patch experiments (left), and then tested how much that decoder could classify a brain activity pattern into Depth or Resolution task in the Natural image experiments (right)^17^.

In the present study, we used an fMRI decoding technique to directly test whether the findings made with Gabor patches can be applied to natural images. We hypothesized that the characteristics of brain activities for visual tasks were independent of the nature of the visual stimuli. Specifically, the cortical responses to the visual stimulus itself will cancel out in the fMRI analysis of two different tasks when the same set of visual stimuli is used. The only reason for a difference in the cortical activation pattern would be some difference in the top–down processing during the two tasks, and the fMRI decoder in the experiment will only be sensitive to differences in the activation caused by the demands of the task. On top of that, focusing on not the fMRI activity contrasts but activity pattern differences would give us more accurate classification to grasp the relationship between spatial pattern of fMRI activity and assigned tasks^7,16^. Based on this hypothesis, we evaluated whether or not the task-dependent fMRI decoder constructed from Gabor patch experiments could predict what task a participant was engaged in natural image experiments (Fig. 2).

As the two different visual tasks, we used psychophysical experiments from previous studies, namely the Resolution and Depth tasks, which examine the relation between display resolution and depth sensation^17–19^. In the Resolution task, participants choose one of two visual image having higher resolution upon monocular viewing (Fig. 2). In the Depth task, participants report which visual image provides the greater depth sensation upon monocular viewing (Fig. 2). The same set of visual stimuli was used in both the Resolution and Depth tasks. In previous studies, it was found that higher resolution facilitated a depth sensation in both Gabor patches and more natural images^17–19^. If the task-dependent fMRI decoder could work independently of the kind of visual stimuli, it might highlight the brain activities with respect to and in connection with the task itself. At the same time, that might indicate that the fMRI decoding technique provides us with a research method for solving the long-standing issue.

**Figure 3:**
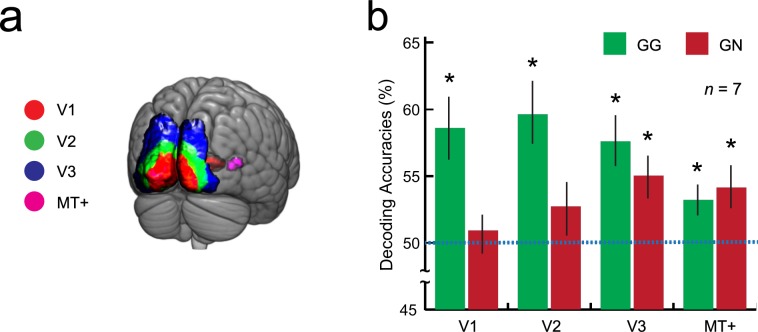
(**a**) Anatomical labels for region of interest analysis, V1, V2, V3, and MT+ were actually used. Posterior view of brain. (**b**) Mean decoding accuracies for GG and GN conditions (n = 7; 192 trials for each participant). Green bars represent the mean task-dependent decoding accuracy for distinguishing Depth or Resolution task with Gabor patch experiments (GG condition, see also the left part of Fig. 2). Red bars represent the mean task-dependent decoding accuracy for distinguishing Depth or Resolution task, from Gabor patch experiments to Natural image experiments (GN condition, also refer to the right illustration at Fig. 2). Blue dashed line represents the chance-level decoding accuracy (50% here). Asterisks indicate statistical significance (t-test with Holm correction). Vertical error bars, ±1 SEM.

As the decoding analyses, we firstly constructed a decoder for each visual area: V1, V2, V3 and the middle temporal area (MT+), which might be principally involved in this kind of shading depth perception and display resolution perception^18,20–26^ (Fig. 3a). We used a linear support vector machine as an implementation of a decoder; this is one of the standard methods in previous fMRI decoding studies^5–9^. The decoder in each visual area classified fMRI patterns into two categories, Depth or Resolution tasks, depending on which the participant was undertaking. Specifically, each decoder conducted the classification by computing a weighted sum of each voxel activity and thresholding the value, e.g., if the value exceeded the threshold, then the fMRI patterns were classified into the Depth task; otherwise, they were classified as the Resolution task (see Methods). The weight and threshold values were determined using the training data set (Fig. 2, left). Therefore, it was expected that even non-informative data analyzed within a single voxel would become informative with multi-voxel patterns^7,16^. In other words, a more sensitive analysis could be expected with the decoding technique than with a conventional single voxel-based analysis, such as general linear models (see Methods). For testing how successful the decoder predicted which task a participant was engaged in, we calculated separate decoding accuracies for each participant and each regions of interest (ROI) (see Methods).

## Results

The psychophysical results in the Gabor patch experiments were consistent with those from previous studies, i.e., a higher resolution stimulus resulted in a stronger depth sensation, even when the participants did not notice the resolution difference^17–19,27^ (Supplementary Information). The fMRI decoding analyses showed that the decoder constructed following the Gabor patch experiments was effective in some visual areas for predicting which task participants were engaged in, i.e., Depth or Resolution task, with Gabor patches (Gabor to Gabor (GG), green bars in Fig. 3b; see Methods). In detail, the mean decoding accuracies were significantly higher than the chance-level (50% here) in V1, V2, V3, and MT+. More importantly, it was found that the fMRI decoder generated using the Gabor patch experiments was partially effective for natural image experiments, especially in V3 and MT+ (Gabor to natural image (GN), red bars in Fig. 3b; see Methods).

The results of the current study demonstrate two important things. First, task-dependent fMRI decoding allows us to directly test the degree to which findings made with Gabor patches can be applied to natural images. This suggests that fMRI decoding is a potential research method for solving a long-standing question in psychology: how much of the simple findings in a laboratory can be applied to everyday complicated psychological events?

Secondly, the task-dependent decoder formed from the Gabor patch experiments was applicable for both Gabor patches and natural image experiments in V3 and MT+. This indicates that these areas play a critical role in depth perception rather than the perceptual processing of display resolution. This is inherently reasonable, because V1 and/or V2 are thought to be involved in more primary perceptual processing such as luminance-contrast perception^2^ (in this case, nearly-perceptual processing of display resolution). In fact, this finding is consistent with previous studies^18,20–26^, which indicate that the fMRI decoding technique is highly reliable for fundamental research with a primitive visual stimulus as well as in more general situations. In the current study, we also tested the decoding accuracy in the lateral occipital complex (LOC) next to MT+^28^, but this was not effective in the GG condition (see Supplementary Information). However, we believe this is, because the main role of LOC is object recognition^28^. Depth and Resolution tasks with Gabor patches in this study do not require object recognition.

One interpretation of the current results is that the higher order processes involved in the respective tasks contributes to the cortical activation of certain visual areas (GG in V1, V2, V3, and MT+, GN in V3 and MT+), whereas they do not contribute to others (GN in V1 and V2, GG and GN in LOC). However, the nature of such top-down processing is unclear at this moment, which makes a direct comparison between GG and GN difficult. Perhaps some brain regions that play a task-dependent top-down modulation role are involved in the processing (e.g., dorsolateral prefrontal cortex (DLPFC))^9,29–31^. Actually, we analyzed the decoding accuracy in DLPFC, but it was shown that it failed to classify the brain activity pattern into one of the two tasks, Depth or Resolution task in this study (see Supplementary Information). To clarify the contributions from top-down control and interactions between the higher-level processing and lower-level visual areas, further experiments and analyses are needed.

Although we obtained significant accuracy values from the decoding experiments, they were not especially high (highest values were 60–65%.). At this point, further research is required to establish more effective fMRI decoding methods. To construct a more accurate decoder and perform detailed analysis, increasing the number of training sessions or using functional, rather than structural, ROI may be helpful. Additionally, NG (natural image to Gabor patch) as well as GN decoding should have been tested to double-check the results and the versatility of the current findings^32^. Our present data do not allow us to check the decoding accuracy of the NG condition easily because there are insufficient number of trials to construct the decoder with natural images (see Methods). However, this possibility must be tested in future studies.

Overall, the current results are firm evidence that the fMRI decoding technique represents an excellent tool for applying laboratory findings to more general situations, as well as providing new insights into the mechanism of shading depth perception.

## Methods

### Participants

Seven participants, aged 20–39 years, and having normal or corrected vision, conducted in a series of experiments, including control experiments. They used their right eye and right hand for the tasks. All participants gave written informed consent and the study was approved by the Ethics Committee of the NHK Science and Technology Research Laboratory, and was performed in compliance with the Declaration of Helsinki.

### Apparatus

A 27′ IPS-TFT color LCD Monitor (ColorEdge CG275W, EIZO Nanao Corp.) was used to present the stimuli. The display had an area of 2560 × 1440 pixels with a pixel size of 0.2331 mm × 0.2331 mm and the contrast ratio of 850:1. Color calibration was performed before the experiments to correct the color balance and display gamma. We applied a gamma of 2.2 and used 256 gray levels (8-bit color depth) to present the stimuli. Visual stimuli were presented using Psychtoolbox 3 (Psychophysics Toolbox, RRID:rid_000041) on Windows 7. The distance between the participants and the display was 5.69 m.

### Stimuli and procedure

In both the Depth and Resolution task with Gabor patches (Training Session), two stimuli with the same size but different resolutions were shown in a vertical orientation for a total of 5 s in a trial (stimuli were presented five times for 1 s, with an interval of 1 s between each showing). A response time of 4 s was available immediately after stimulus had been presentated. We set three differnt stimuli, with 30, 60, or 120 cycle per degree (cpd), and three orientations, 0° (perpendicular), 45°, or 135°. The original Gabor patch was as follows: Spatial frequency of the Gabor was 6.2 cycles /degree. The background was gray, 13.9 cd/m^2^. The maximum luminance of the Gabor was 46.3 cd/m^2^, and the minimum was 3.9 cd/m^2^. The processes to downconvert were as follows: A low-pass filter was applied to the image in spatial domain with the cut-off frequency of 0.5 (-6 dB at cut-off frequency) in normalized spatial frequency. The filtered image was re-sampled by a factor of 1/2 in both row-wise and column-wise, resulting in a half-size image to the original one. After that, each pixel of the image was replicated and interpolate among pixels to create same size but low-resolution images. These processes were applied to the images of 120 cpd (original image) to create the images of 60 cpd, and also applied to the images of 60 cpd to create the images of 30 cpd. We set two resolution combinations of 120 cpd to 60 cpd or 30 cpd to 60 cpd (we did not tell this to participants). The position of stimuli randomly assigned to top or bottom across trials. Each set was repeated 4 times in one session, so that one session consisted of 2 (resolution combination) × 3 orientations × 4 repetitions = 24 trials (336 seconds). There were 8 sessions (4 Depth task sessions + 4 Resolution task sessions). Therefore, the total number of trials was 192.

For the natural image experiments (Test Session), the drawings were created by Y.S and Y.T., and modified using Adobe Illustrator CC (Fig. 1b). The down-conversion processing, resolutions, and experimental design were identical to those for the Gabor patch experiment except that there were only two sessions (one Depth and one Resolution task session).

## MRI Data acquisition

MRI data were obtained using a 3 T MRI scanner (MAGNETOM Trio A Tim; Siemens, Erlangen, Germany) using a standard head coil at the ATR Brain Activity Imaging Center (Kyoto, Japan). An interleaved T2*-weighted gradient-echo planar imaging (EPI) scan was performed to acquire functional images to cover the entire brain (TR, 2000 ms; TE, 30 ms; flip angle, 80°; FOV, 192 mm × 192 mm; voxel size, 3.5 mm × 3.5 mm × 4.0 mm; slice gap, 1 mm; number of slices, 30). T2-weighted turbo spin echo images were scanned to acquire high-resolution anatomical images of the same slices used for the EPI (TR, 6000 ms; TE, 57 ms; flip angle, 90°; FOV, 256 × 256 mm; voxel size, 0.88 mm × 0.88 mm × 4.0 mm). T1-weighted magnetization-prepared rapid-acquisition gradient echo (MP-RAGE) fine-structural images of the whole head were also acquired (TR, 2250 ms; TE, 3.06 ms; TI, 900 ms; flip angle, 9°; FOV, 256 mm × 256 mm; voxel size, 1.0 × 1.0 × 1.0 mm).

## MRI Data Analyses

We used anatomical labels for ROI analysis of V1, V2, V3, and MT+, anatomically defined by the SPM anatomy toolbox (RRID:nif-0000-10447). LOC and DLPFC were determined in different ways (see Supplementary Information). Because the ROIs obtained from the toolbox were defined in the standard brain, we converted them into individual brains with the ‘deformation’ tool implemented in SPM5. The first 6 seconds scans of each sessions were discarded to avoid the effect of instabilities in the MRI scanner. The acquired fMRI data underwent slice-timing correction and three-dimensional motion correction using SPM5. The data were then coregistered to the within-session high-resolution anatomical image of the same slices used for EPI and subsequently to the whole-head high-resolution anatomical image. The coregistered data were reinterpolated as 3.5 mm × 3.5 mm × 5.0 mm voxels.

To elucidate the engagement of localized brain regions in the tasks, we created data samples consisted of fMRI activity patterns in each ROI, constructed decoders to classify the data samples according to the task in which a participant was undertaking, and computed prediction accuracies for each decoder. Specifically, the data samples were created by averaging the fMRI volumes within each 10 s stimulus block (average of five volumes, shifted by four seconds to account for hemodynamic delays), normalizing voxel activities relative to the average of the entire time course within each session, and sorting voxels into each ROI. And then, the data samples were labeled with the task (Depth or Resolution) and used as input for the decoders.

The classification was conducted by computing a linearly weighted sum of fMRI voxel activities (values in each element in a data sample represented as a vector) and by thresholding the value. The weights and a threshold value were determined using a training data set based on the support vector machine algorithm^33^. For prediction, when the value exceeded the threshold, then the data sample was classified into the Depth task; otherwise, it was classified into the Resolution task. In fact, the computation performed by a task decoder for each ROI can be represented by a linear function of an input data sample, or voxel activities, ${\bf{x}}=({x}_{1},{x}_{2},\mathrm{..}.,{x}_{d})$:

$${f}_{{\rm{ROI}}}({\bf{x}})=\mathop{\sum }\limits_{i=1}^{d}{w}_{i}{x}_{i},$$

where ${w}_{i}$ is the weight for *i*-th element (single voxel activity) of a data sample and *d* is the number of all voxels in the ROI. To obtain the prediction of the engaged task, we compared the output of the function with a threshold ${w}_{0}$. When ${f}_{{\rm{ROI}}}({\bf{x}}) > {w}_{0}$, the decoder predicted that **x** was obtained while the participant was engaged in the Depth task, otherwise in the Resolution task. In this study, we used a linear classification library, liblinear, as a support vector machine implementation, with the default value of a soft margin parameter (*C* = 1) in the parameter settings^34^. Finally, we computed confusion matrices and obtained prediction accuracies for each decoder and participant. If the mean classification accuracies for a ROI across participants was significantly higher than the chance rate, the selected ROI should be engaged in the neural processing of the Depth and/or Resolution tasks.

The decoders were trained with data samples from the six Gabor-patch sessions (three sessions each for Depth and Resolution tasks) out of the total of eight sessions, and tested with data samples from the remaining two Gabor-patch sessions (GG condition) and the two natural-image sessions (GN conditions), which consisted of a depth and a resolution task session. To compensate for the variability in the choices of a training and test data-sets, the decoding performance was computed in a cross-validation manner whereby training and testing of decoders were performed based on all possible combinations of the Depth and Resolution task sessions.

## Supplementary information

Supplementary Info.

## Data Availability

The data that support the plots within this study and other findings of this study are available in the main text and the Supplementary Information. Additional information is available from the corresponding author upon reasonable request.
