# Supplementary material for: Task-dependent fMRI decoder with the power to extend Gabor patch results to Natural images
Source: Sci Rep. 2020 Jan 28;10:1382. doi: 10.1038/s41598-020-58241-x (PMC6987206; doi:10.1038/s41598-020-58241-x)
Supplement: Supplementary file 1 — Supplementary Info. [file 41598_2020_58241_MOESM1_ESM.docx]

**Supplementary Information:**

**“Task-dependent fMRI decoder with the power to extend Gabor patch results to Natural images.”**

Yoshiaki Tsushima, Yasuhito Sawahata, and Kazuteru Komine

**
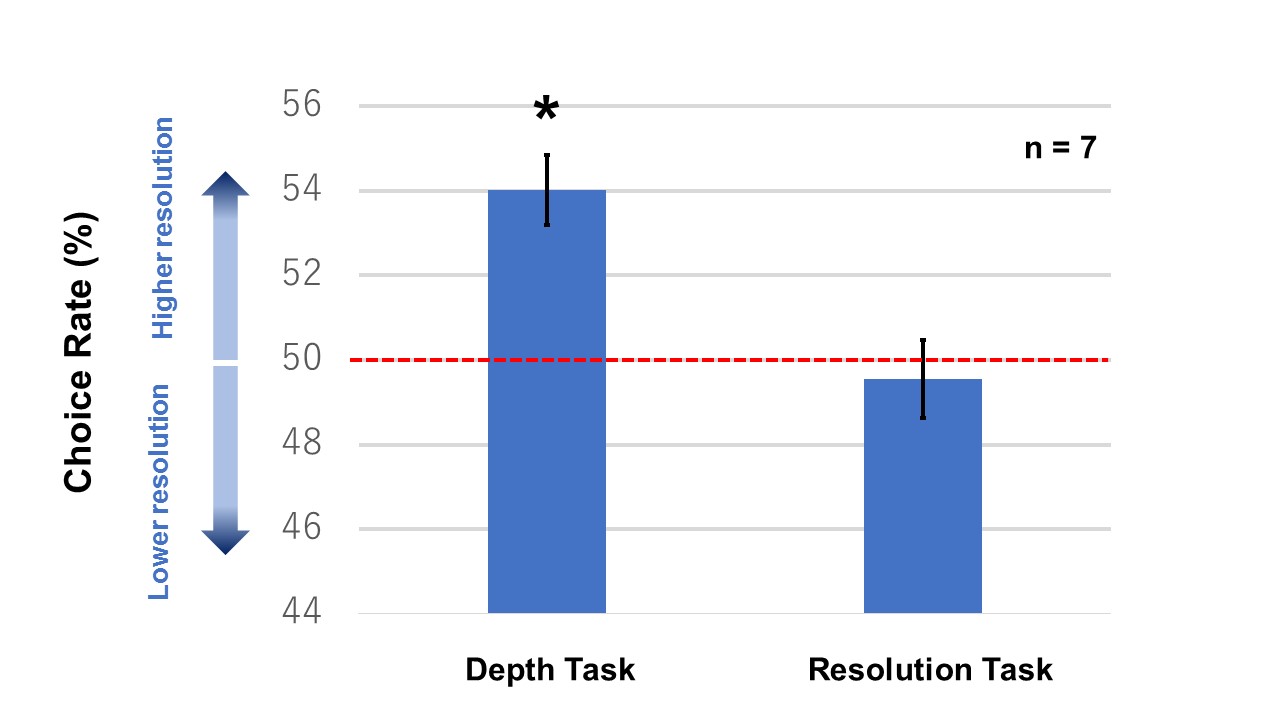
**

**Supplementary Figure 1.** Mean behavioral results of Gabor patch experiment (Training Session). Vertical error bars, ±1 SEM. A red dashed line represents the chance-level choice rate (50%). Although the mean choice rate in Resolution task was not different from chance-level, that in Depth task was significantly higher than chance-level (*n* = 7, *p* < 0.01, against 50%). These results indicate that higher resolution in Gabor patch facilitated depth sensation even when participants did not realize the resolution difference. These results are in accordance with the previous findings^8,9,17^.

**Supplementary Figure 2.** Mean decoding accuracies at lateral occipital cortex (LOC) in GG and GN conditions (n=7; 192 trials for each participant). A blue dashed line represents the chance-level decoding accuracy (50% here). Vertical error bars, ± 1 SEM.

The mean decoding accuracy at LOC was not significantly higher than chance-level (50% here) in GG condition (A green bar; t-test *p =* 0.064). We think it is acceptable, because the main role of LOC is object recognition (Grill-Spector, Kourtzi, and Kanwisher, *Vision Research*, 2001). Depth and Resolution tasks with Gabor patch in this study did not essentially require the processing of object recognition. We could procedurally compute a prediction accuracy in GN condition (a red bar), but the value did not mean anything here because the decoder without a significant accuracy, or a prediction capability, in GG condition could not perform the prediction also in GN condition.

LOC was defined based on the previous study (Song and Jiang, *NeuroImage*, 2006). Since the method to make ROI for analysis was different from other areas in the main text (**See Methods**), we present the results of LOC separately from other results.

**Supplementary Figure 3.** Mean decoding accuracies at dorsolateral prefrontal cortex (DLPFC) in GG and GN conditions (n=7; 192 trials for each participant). A blue dashed line represents the chance-level decoding accuracy (50%). Vertical error bars, ± 1 SEM.

The mean decoding accuracies at DLPFC were not significantly higher than chance-level (50%) in both GG (A green bar; t-test *p =* 0.057) and GN conditions (A red bar; t-test *p =* 0.109).

DLPFC was defined as Broadmann area 46 by Talairach Software ([*www.talairach.org*](http://www.talairach.org)*,* Lancaster et al., *Human Brain Mapping,* 1997, 2000).

**References**

Grill-Spector K., Kourtzi Z., and Kanwisher N. The lateral occipital complex and its role in object recognition. *Vision Research*. **41**: 1409-1422, (2001)

Song, JH. and Jiang Y. Visual working memory for simple and complex feature. *NeuroImage*. **30**: 963-972, (2006)

Lancaster JL, Woldorff MG, Parsons LM, Liotti M, Freitas CS, Rainey L, Kochunov PV, Nickerson D, Mikiten SA, Fox PT, "Automated Talairach Atlas labels for functional brain mapping". *Human Brain Mapping* 10:120-131, (2000)

Lancaster JL, Rainey LH, Summerlin JL, Freitas CS, Fox PT, Evans AC, Toga AW, Mazziotta JC. Automated labeling of the human brain: A preliminary report on the development and evaluation of a forward-transform method. *Hum Brain Mapping* 5, 238-242, (1997)
